# Identification and Characterization of a *PutAMT1;1* Gene from *Puccinellia tenuiflora*

**DOI:** 10.1371/journal.pone.0083111

**Published:** 2013-12-10

**Authors:** Yuanyuan Bu, Bo Sun, Aimin Zhou, Xinxin Zhang, Imshik Lee, Shenkui Liu

**Affiliations:** Key Laboratory of Saline-Alkali Vegetation Ecology Restoration in Oil Field (SAVER), Ministry of Education, Alkali Soil Natural Environmental Science Center, Northeast Forestry University, Harbin, P.R.China; Ghent University, Belgium

## Abstract

Nitrogen is one of the most important limiting factors for plant growth. However, as ammonium is readily converted into ammonia (NH_3_) when soil pH rises above 8.0, this activity depletes the availability of ammonium (NH_4_^+^) in alkaline soils, consequently preventing the growth of most plant species. The perennial wild grass *Puccinellia tenuiflora* is one of a few plants able to grow in soils with extremely high salt and alkaline pH (>9.0) levels. Here, we assessed how this species responds to ammonium under such conditions by isolating and analyzing the functions of a putative ammonium transporter (PutAMT1;1). PutAMT1;1 is the first member of the AMT1 (ammonium transporter) family that has been identified in *P. tenuiflora*. This gene (1) functionally complemented a yeast mutant deficient in ammonium uptake (2), is preferentially expressed in the anther of *P. tenuiflora*, and (3) is significantly upregulated by ammonium ions in both the shoot and roots. The PutAMT1;1 protein is localized in the plasma membrane and around the nuclear periphery in yeast cells and *P. tenuiflora* suspension cells. Immunoelectron microscopy analysis also indicated that PutAMT1;1 is localized in the endomembrane. The overexpression of PutAMT1;1 in *A. thaliana* enhanced plant growth, and increased plant susceptibility to toxic methylammonium (MeA). Here, we confirmed that PutAMT1;1 is an ammonium-inducible ammonium transporter in *P. tenuiflora*. On the basis of the results of PutAMT1;1 overexpression in *A. thaliana*, this gene might be useful for improving the root to shoot mobilization of MeA (or NH_4_^+^).

## Introduction

Nitrogen is one of the essential macronutrients required for the synthesis of many cellular components, including amino acids, proteins, chlorophyll, nucleic acids, lipids, and a variety of other metabolites containing nitrogen in their structure [1]. Nitrogen is also one of the most important nutrients for crop production; however, the total nitrogen content of agricultural soils is reduced by salinization, the current irrigation agricultural causes soil salinization [2,3]. Of the two main nitrogen ions present in soil, ammonium (NH_4_^+^) and nitrate (NO_3_^-^), ammonium is the preferential form for nitrogen uptake in plants subject to nitrogen deficiency [4], because less energy is required for ammonium assimilation compared to nitrate assimilation [5]. However, excess NH_4_^+^ might be toxic to plants [6,7]; thus, NH_4_^+^ transport must be regulated. Ammonium uptake in plants is mediated by either nonselective ion channels, such as potassium channels or members of the aquaporin family [8-11], or specific channels belonging to the ammonium transporter (AMT) family [12-14]. 

Physiological studies focusing on NH_4_^+^ uptake in plant roots identified 2 NH_4_^+^ transport systems; a high-affinity transport system (HATS) and a low-affinity transport system (LATS). Subsequent phylogenetic studies indicated that the plant transporters of the AMT family are subdivided into 2 subfamilie: AMT1 and AMT2 [15]. The former family encompasses either NH_4_^+^ uniporters or NH_4_^+^/H^+^ co-transporters [15–18]. Normally, AMT1 subfamily members are expressed in the plant roots [19], and are generally repressed by high nitrogen levels [20]. AMT1 family genes have been shown to regulate root ammonium fluxes in response to cellular and/or whole-plant demand for nitrogen [4,21,22]. To date, several AMT genes have been isolated in plants that differ in their tissue specificity and in their inducibility by nitrogen availability. All 3 rice genes (OsAMT1;1–3) express high affinity transporter activity, with northern blot analysis showing 3 distinct patterns of expression; (1) OsAMT1;1 is constitutively expressed in both the roots and shoots of rice; (2) OsAMT1;2 expression is root specific and ammonium inducible; and (3) OsAMT1;3 expression is root specific and nitrogen depressible [21,23]. AtAMT1;1, AtAMT1;3, and AtAMT1;5 are expressed in the rhizodermal cells of the root hair zone of *Arabidopsis*, AtAMT1;2 is localized to the endodermal and cortical cells [13,14], and AtAMT1;4 seems to be expressed in the pollen grains and pollen tube [24]. The localization of one AMT gene (OsAMT1;2) in the central cylinder and cell surface of the root tips of rice was confirmed by *in-situ* mRNA hybridization [23]. At the cellular level, all of the membranes of plant AMT proteins studied to date have been found to be localized to the plasma membrane [13,14,17,25,26]. 

Recently, the physiological role of *Arabidopsis* AMT1 family members has been intensively investigated in roots. It was found that AtAMT1;1, AtAMT1;3, and AtAMT1;5 contribute additively to approximately 70–80% of the total high-affinity ammonium uptake capacity of roots [13,14]. AtAMT1;4 also mediates ammonium uptake into plant roots. However, two-fold higher ammonium uptake was recorded for transgenic lines of AtAMT1;4 grown on medium containing different nitrogen sources compared to mutant lines [24]. Thus, existing studies confirm that the AMT1 gene family regulates root ammonium fluxes in response to cellular and/or whole-plant demand for nitrogen.

As ammonium is readily converted into ammonia (NH_3_) when soil pH rises above 8.0, ammonium availability in alkaline soils is often depleted, consequently preventing the growth of most plant species [27]. However, information about the transport mechanisms and the nature of the transported substrates (NH_3_ and NH_4_^+^) remains limited. Alkaline soil, which is a type of soil that is quite common in northern China, contains high levels of sodium carbonate and sodium bicarbonate (soda) [28,29], the hydrolytic decomposition of which raises soil pH to above 9.0. In contrast to many other plant species unable to cope with the nutrient limitations of alkaline soils, Puccinellia tenuiflora is a monocotyledonous halophyte species that thrives under the high pH conditions (pH≈10) of the Songnen plain in northeastern China. These plains are characterized by extreme nitrogen shortages in the soil environment. Thus, in this study we set out to elucidate the mechanism of ammonium transport in the halophyte P. tenuiflora under these extreme conditions.

In this work, a putative AMT from P. tenuiflora was named PutAMT1;1 (GenBank accession NO: JQ279059). This gene was studied to obtain preliminary insights about how this species survives in extreme saline alkali soil at pH 10 under conditions of nitrogen and H^+^ shortage. The tissue-specific expression of this gene was studied, along with its responsiveness to ammonium. The localization of PutAMT1;1 was determined in yeast and cell suspensions of *P. tenuiflora* using a GFP marker and immunoelectron microscopy analysis. The subcellular localization of PutAMT1;1 in *P. tenuiflora* differed compared to that in previously reported plant species. The *PutAMT1;1* gene was functionally characterized through the complementation of a mutant yeast strain deficient in nitrogen uptake and through overexpression in *Arabidopsis* grown in media containing different nitrogen sources and methylammonium (MeA).

## Materials and Methods

### Strains and plant materials

The *Saccharomyces cerevisiae* mutant strain 31019b (*Δmep1Δmep2:*:LEU2 *Δmep3:*:KanMX2 *ura3;* Marini *et al*., 1997) is defective in the expression of 3 endogenous AMT genes (mep1, mep2, and mep3) [30]. This strain is unable to grow on medium containing less than 5 mM (NH_4_)_2_SO_4_ as a sole nitrogen source. The yeast strain INVSc1 and the *Agrobacterium tumefaciens* strain EHA105 were used to express GFP fusion proteins in yeast and plants, respectively. P. tenuiflora plants were collected from an area of alkaline soil in North-East China (Heilongjiang Province). No specific permission were required for these locations/activities, the area of alkaline soil in North-East China are the public open place, and the sample activities did not involve any endangered or protected species. *Arabidopsis* (*Arabidopsis thaliana*) ecotype Columbia-0 was used for overexpression of PutAMT1;1. The plants in which overexpression was identified were used for RNA extraction and northern hybridization. 

### Plasmid constructs

The open reading frame (ORF) of PutAMT1;1 was amplified from PutAMT1;1 (JQ279059) cDNA using the primers *PutAMT1;1-FW* and *PutAMT1;1-RV* (Table 1). The amplified product was digested with *Eco*RI and *Xba*I, and cloned into the yeast expression vector pYES2 (*Invitrogen*) to form *pYES2-PutAMT1;1* plasmid, which was confirmed by sequencing. This construct was used for yeast complementation.

**Table 1:** Details of primers used for polymerase chain reaction analysis.

Primer name	Sequence
*PutAMT1;1-FW*	5′-GAATTCATGTCGACGTGCGCG-3′(*Eco*RI)
*PutAMT1;1-RV*	5′-TCTAGACTAGACGTGGCCGCCA-3′(*Xba*I)
*GFP- FW*	5′-AAGCTTATGTCGACGTGCGCG-3′
*GFP- RV*	5′-GGATCCACGTGGCCGCCAT-3′
*pBI121-PutAMT1;1-FW*	5’-TCTAGAATGGTGAGCAAGGG-3’ (*Xba*I)
*pBI121-PutAMT1;1-RV*	5’-GAGCTCCTTGTACAGCTCGTCCATG-3’(*Sac*I)
*Semi-RT-FW*	5′-CTCATCGGGCTCAACACGGT-3′
*Semi-RT-RV*	5′-CGCTGACCTGAGCATGAACG-3′
*Actin-FW*	5′-GTGTCAGCCATACTGTGCCAATC-3′
*Actin-RV*	5′-TTGCTCATGCGGTCAGCAATACC-3′
*Real-time -FW*	5′-GAACATCATGCTCACCAACG-3′
*Real-time- RV*	5′-GGGAATGTCTCGAAGACCAA-3′
*Tubulin-FW*	5′-GCTGACCACACCTAGCTTCGGGG-3′
*Tubulin-RV*	5′-GACCAGGGAACCTCAGGCAGC-3′

For the construction of GFP fusion proteins, the GFP gene without the stop codon was amplified with the primers *GFP-FW* and *GFP-RV* (Table 1) by using the pEGFP vector as a template. The PCR product was digested with *Hin*dIII and *Bam*HI, and then cloned with the plasmid *pYES2-PutAMT1;1* to obtain the plasmid *pYES2-PutAMT1;1-GFP*.

*pBI121-GFP* and *pBI121-PutAMT1;1-GFP* constructs for plant transformation were completed using the following methodology. First, the PCR products were amplified using plasmid *pYES2-PutAMT1;1-GFP* as a template with primers *pBI121-PutAMT1;1*-FW and *pBI121-PutAMT1;1*-RV (Table 1), respectively, then these products were further digested with *Xba*I and *Sac*I, and ligated into the vector pBI121, which was also digested with *Xba*I and *Sac*I.

### Phylogenetic analyses, yeast transformation, and growth conditions

Full-length amino acids sequences were aligned using CLUSTALX, and imported into the Molecular Evolutionary Genetics Analysis (MEGA) package version 3.1 [31]. Phylogenetic analyses were conducted using the neighbor joining (NJ) method implemented in MEGA. The following accession numbers were used: PutAMT1;1 (GenBank accession number: JQ279059), AtAMTl;1 (CAA53473), AtAMTl;2 (AAD54639), AtAMTI;3 (AAD54638), AtAMTl;4 (CAB81458), AtAMTl;5 (NP_189072), AtAMT2 (NP_181363), OsAMTl;1 (AAL05612), OsAMTl;2 (AAL05613), OsAMTl;3 (AAL05614), OsAMT2;1 (BAB87832), OsAMT2;2 (NM_190445), OsAMT2;3 (NM_190448), OsAMT3;1 (BAC65232), OsAMT3;2 (AAO41130), OsAMT3;3 (AK108711), OsAMT4 (AAL59860), OsAMT5 (ABA95601), LjAMT1;1 (AF182188), LjAMT1;2 (AAM95453), LjAMT1;3 (CAE01484), LjAMT2 (AF187962), LeAMT1;1 (X92854), LeAMT1;2 (X95098), LeAMT1;3 (AF118858), BnAMT1;1 (AY642429), BnAMT1;2 (AF306518), TaAMT1;1 (AAS19466), TaAMT1;2 (AAS19467), TaAMT1;3 (AAR27052), and TaAMT2 (AAR87397).

Yeast transformation was performed using a lithium acetate-based method [32]. The plasmid *pYES2-PutAMT1;1* and *pYES2* empty vector were used as controls, and were introduced into yeast strain 31019b. For complementation assays, the yeast transformants were cultured in liquid SD-Uracil medium until OD600≈1, diluted × 10^-1^–10^-4^, and dropped onto solid yeast nitrogen base (YNB) medium (without amino acids or ammonium sulfate) that was supplemented with 0, 1, 5, 10, and 20 mM (NH_4_)_2_SO_4_. For the response assays of yeast cells to different pH levels, yeast transformants were grown on YNB medium containing 1 mM (NH_4_)_2_SO_4_, with the pH being adjusted to 4.0, 5.0, 6.0, 7.0, and 8.0. All of the plates were incubated at 30°C for 3–5 days before conducting growth assessments.

### Analysis of gene expression using real-time PCR

*P. tenuiflora* plants were cultured for 3 weeks, then the leaf, root, stem, panicle, panicle stem leaf sheath, and anther were sampled to determine the patterns of expression using semi-quantitative RT-PCR analysis. For the analyses of expression levels, *P. tenuiflora* plants were cultured for 3 weeks, then transferred to nutrient solution containing 500 µM (NH_4_)_2_SO_4_, Ca(NO_3_)_2_, or NH_4_NO_3_ as the sole nitrogen source. For all treated plants, as well as for the corresponding controls without nitrogen, the shoots and roots were sampled at 2 and 24 h after treatment. All samples were frozen in liquid nitrogen and stored at -80°C until use. 

Total RNA was isolated using the RNeasy plant Mini kit (*Qiagen, Germany*), and treated with RNase-free DNaseI (*Qiagen, Germany*). First-strand cDNA was synthesized using SuperScript III reverse transcriptase (*Invirogen,USA*). Gene-specific primers were used as references for semi-quantitative RT-PCR analysis; namely, *Semi-RT-*FW and *Semi-RT-*RV (Table 1) for PutAMT1;1 and *Actin-FW* and *Actin-RV* for Actin. For real-time PCR analysis, the tubulin gene from *P. tenuiflora* was cloned and used as an internal standard for the PutAMT1;1 gene. The primer pairs *Real-time-*FW and *Real-time-*RV (Table 1) were used for PutAMT1;1, while *Tubulin-FW* and *Tubulin-RV* (Table 1) were used for tubulin. Relative quantification using real-time PCR reactions were performed with SYBR green I using the LightCycler^®^480 system II (*Roche USA*).

### Localization of PutAMT1.1 protein in yeast and plant cells

The plasmids *pYES2-PutAMT1;1-GFP* and *pYES2-GFP* were introduced into the yeast strain INVSc1. These yeast transformants were pre-cultured in liquid Yeast Extract Peptone Dextrose (YPD) medium overnight at 30°C, and washed 3 times with distilled water. The cells were cultured in liquid SD-Uracil medium with galactose at 30°C to induce the expression of GFP and PutAMT1;1-GFP. *Arabidopsis* and P.tenuiflora suspension cells previously electroporated with plasmids *pBI121-GFP* or *pBI121-PutAMT1;1-GFP* were transformed with *Agrobacterium tumefaciens. Arabidopsis* protoplasts were prepared following that previously described by Sheen (2002) [33]. The resulting GFP and chloroplast channel images were overlaid. P. tenuiflora suspension cells that had been previously electroporated with the plasmids *pBI121-GFP* and *pBI121-PutAMT1;1-GFP* were transformed with *Agrobacterium tumefaciens* to obtain transgenic P. tenuiflora cell lines. Five-day-old P. tenuiflora suspension cells were used for protoplast preparation. Subsequently, the cell wall was digested for 3–6 h at 28°C by incubating it in 1 ml enzymatic mixture containing 1% cellulase (*Sigma,USA*) and 1% pectinase (*Sigma,USA*) in plasmolysis medium supplemented with 0.1% bovine serum albumin (BSA). The detection of all GFP signals was carried out using a laser-scanning confocal imaging system (*Olympus Fluoview, FV500*).

### Immunogold electron microscopy localization

Five-day-old seedlings grown on vertical plates were chemically fixed in 4% paraformaldehyde and 2.5% glutaraldehyde (for root tissues) in 0.1 M phosphate buffer (pH7.4) under vacuum (4 h). The plant tissue was dehydrated and infiltrated with London Resin White LRW (*Sigma, USA*), polymerization was carried out at 60°C. Ultrathin sections of 60–90nm were cut using an EM UC6 ultramicrotome (*Leica, Germany*), and placed on formavar-coated nickel grids. Immuno-labeling was performed on these samples, using a primary antibody (mouse anti-GFP, *Roche, USA*) diluted at a ratio of 1:200. The samples were then left overnight at 4°C. This step was followed by rinsing and incubation with secondary antibodies (rabbit anti-mouse, *Sigma, USA*) that had been conjugated to 10-nm gold particles. Secondary antibodies were diluted in Phosphate Buffered Saline (PBS) at a ratio of 1:20, and supplemented with 1% BSA and 2% Tween-20. The samples were then incubated for 2 h at room temperature, and then silver-enhanced for 15 min (Silver lactate, *Sigma, USA*). Sections were post-stained with aqueous urany1 acetate, and examined under an H-7500 transmission electron microscope (*Hitachi, Japan*) operating at 80 kV.

### Northern blot analysis

Total RNA was obtained using Trizol. Denaturing gel electrophoresis was carried out to examine the transformation of the *PutAMT1;1* gene, which was marked with a probe labeled with digoxigenin (DIG, *Roche, USA*) with RNA gel blot analysis, following the methods of Sambrook et al. (1989) [34]. Signals were detected using a luminescent image analyzer (*Fijifilm, LAS-4000mini, Japan*).

### Characterization of transgenic Arabidopsis plants

The construct *pBI121-PutAMT1;1* and the *pBI121* empty vector were transformed into *Arabidopsis* (wild-type, WT) via *Agrobacterium tumefaciens* using the floral dip method [35]. The seeds of *A. thaliana* Col-0 and CaMV35S:PutAMT1;1 over-expression lines were surface-sterilized, and grown on half strength MS medium (1/2 MS) plates containing 1% sucrose, 0.8% agar, and 5 mM nitrate as the sole nitrogen source. The plants were pre-cultured for 7 days, and then transferred to vertical plates containing 1/2 MS medium or were supplemented with different amounts of ammonium succinate at concentrations of 0 µM, 25 μM, and 250 μM as the sole nitrogen source for 7 days. For the toxicity analyses, WT and transgenic plants were also transferred to medium containing 0, 10, and 20 mM MeA in the presence of 1 mM nitrate for 7 days. The plants were all grown in a climate-controlled greenhouse (16/8 h light/dark, temperature 22°C/18°C). Statistical analyses were carried out using the Student’s *t*-test.

## Results

### Cloning and characterization of PutAMT1;1

**Figure 1:**
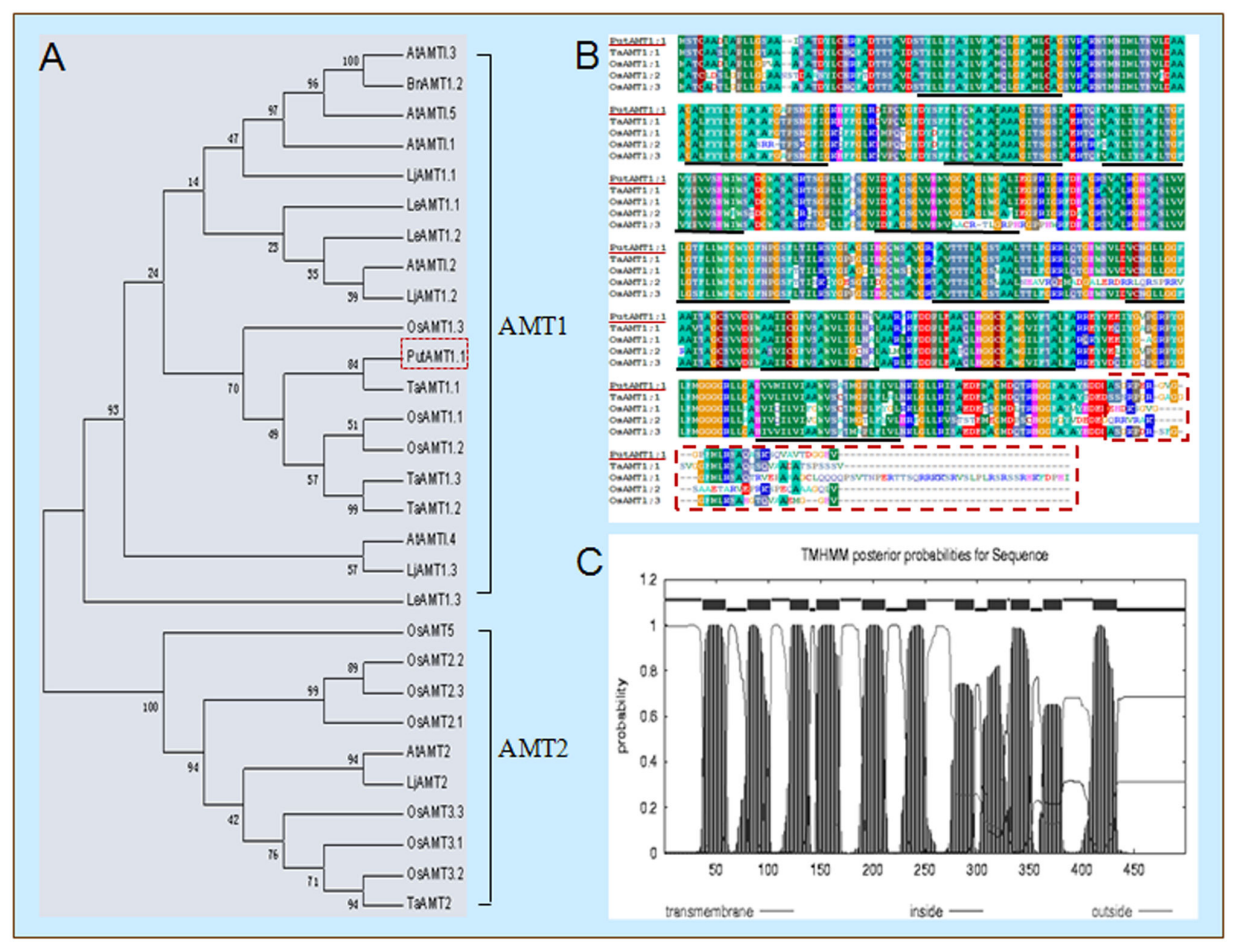
Sequence and phylogenetic analysis of PutAMT1;1. (A) Amino acid sequence of PutAMT1;1 and phylogenetic trees analysis of AMT families; Sequences were obtained from the GeneBank database. The accession numbers are listed in the Material and Methods section. (B) An alignment of the amino acid sequence of PutAMT1;1 (GenBank NO. JQ279059) with TaAMT1;1 (GenBank NO. AY525637), OsAMT1;1 (GenBank NO. AF289477), and AtAMT1;1 (GenBank NO. NM_-_117425); the colored box indicates conserved amino acid residues. (C) The transmembrane domains in PutAMT1;1 were predicted by the TMHMM algorithm (http://www.cbs.dtu.dk/services/TMHMM), and are underlined in black in (B).

The PutAMT1;1 gene (GenBank accession NO: JQ279059) was 2129 bp in length, and was identified from a salt stress *P. tenuiflora* cDNA library. The open reading frame (ORF) was 1500 bp, and encoded a protein of 499 amino acids, with a predicted molecular mass of 55.47 kDa and a predicted *pI* of 5.28. Phylogenetic analysis was performed based on the amino acid sequence of AMT1 and AMT2 subfamily members from different plant species, including *Arabidopsis thaliana*, *Oryza sativa*, *Lycopersicon esculentum*, *Triticum aestivum*, *Lotus japonicus*, *Brassica napus*, and *P. tenuiflora*. Figure 1A shows that PutAMT1;1 belongs to the AMT1 subfamily, and that it is most closely related to the AMT TaAMT1;1, with high affinity.

The PutAMT1;1 polypeptide was 93%, 91%, 88%, and 87% identical to TaAMT1;1 (GenBank NO. AY525637), OsAMT1;2 (GenBank NO. AF289478), OsAMT1;3 (GenBank NO.AF289479), and OsAMT1;1 (GenBank NO. AF289477) proteins, respectively (Figure 1A). PutAMT1;1 is highly conserved with other AMT1 members, with the exception of the C-terminal sequence (yellow box in Figure 1B).

The number and topology of predicted PutAMT1;1 transmembrane domains (11 transmembrane domains; with an N terminus and a C terminus on the exterior and interior face of the membrane, respectively) are similar to the those of other AMT1 polypeptides from *Arabidopsis*, tomato, and yeast [4,30,36] (Figure 1C). Overall, these results indicate that PutAMT1;1 is the first member of the AMT1 family to be identified in *P. tenuiflora*.

### *G*ene expression pattern of PutAMT1;1 under different nitrogen source conditions

**Figure 2:**
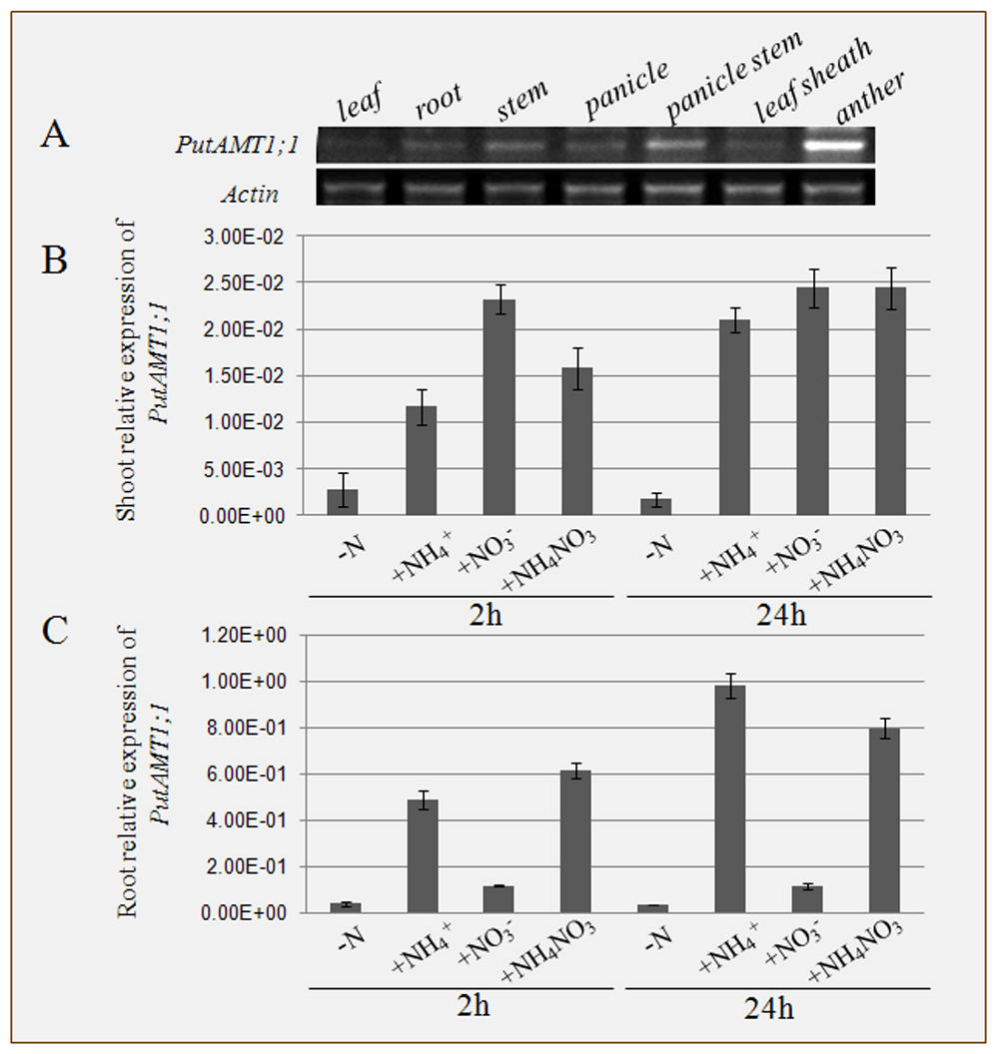
Expression analysis of the *PutAMT1;1* gene. Expression was analyzed using semi-quantitative RT-PCR (A) and real-time PCR in the roots (B) and shoots (C) using actin and tubulin mRNA as reference material, respectively. The expression of PutAMT1;1 is given relative to tubulin mRNA levels, and is the mean of 3 replicates ±S.E. PutAMT1;1 expression in control plants (-N) and plants exposed to different nitrogen sources: (NH_4_)_2_SO_4_, Ca(NO_3_)_2_, and NH_4_NO_3_ at 2 and 24 h after treatment.

To investigate the pattern of PutAMT1;1 expression under normal conditions, RNA was extracted from the leaf, root, stem, panicle, panicle stem, leaf sheath, and anther of plants grown for 3 weeks. Semi-quantitative RT-PCR analyses carried out on various tissues showed that PutAMT1;1 is expressed in all *P. tenuiflora* plant organs, with the expression being higher in the panicle stem and the anther (Figure 2A). These results indicate that PutAMT1;1 expression is developmentally controlled, and that its expression levels increase after the transition from the vegetative to reproductive phase. 

However, interestingly, PutAMT1;1 expression was also found to respond specifically to different nitrogen sources in both the roots and shoots. This experimental design assessed the effect of nitrate and ammonium on PutAMT1;1 expression for time course (as shown in the Materials and Methods). The separate sampling of shoots and roots showed that PutAMT1;1 could be detected following ammonium treatments in either tissue type; however, the roots expressed a much higher absolute level of PutAMT1;1 compared to the shoots (note the different scales in Figure 2B and 2C). Under nitrogen starvation, PutAMT1;1 was expressed at low levels in both tissues, with a further decrease observed in the shoot at 24 h after treatment.

**Figure 3:**
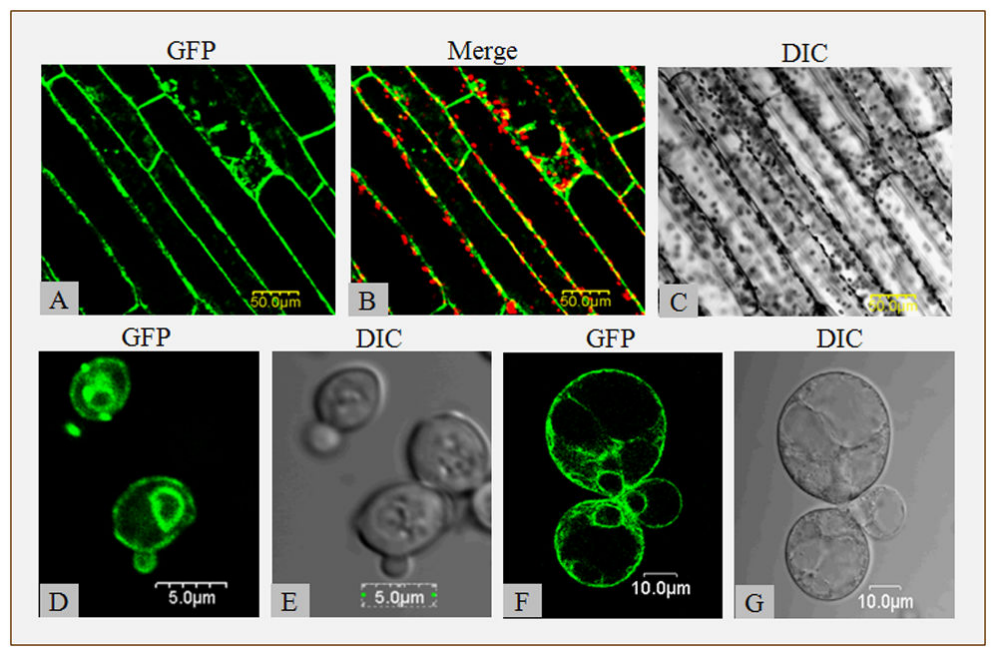
Subcellular localization of PutAMT1;1. (A to C) Using the petiole epidermis of transgenic plant with *pBI121-*PutAMT1;1-GFP. (A) is GFP, (B) is the green GFP fluorescence merged with the chloroplast fluorescence, (C) is DIC; (D to G) GFP fusion proteins of PutAMT1;1 expressed in yeast (D, E) and *Puccinellia tenuiflora* cells (F, G). All images were created using a laser-scanning confocal imaging system (*Olympus* *Fluoview*, *FV500*). GFP fluorescence was excited using an argon laser (488 nm). Chloroplast fluorescence was detected at 620 nm. The size of the scale bars are shown on the images.

Under limited nitrogen availability, a different pattern of PutAMT1;1 expression was observed in the 2 tissue types, both in terms of quality and quantity. In the shoot, the absence of nitrogen repressed the expression of PutAMT1;1, while the presence of nitrate alone did not affect the expression level of the PutAMT1;1 gene. The PutAMT1;1 gene was strongly induced by nitrogen, especially in the roots, was instead observed when ammonium was added to the growth medium either as (NH_4_)_2_SO_4_ or NH_4_NO_3_ (Figure 3A and 3B). Overall, these results indicate that *in vivo* PutAMT1;1 expression is specifically coupled to the concentration of ammonium ions in *P. tenuiflora* cells. 

### Subcellular localization of PutAMT1;1:GFP fusion protein in yeast and plant cells

Using the petiole epidermis of the transgenic plant with *pBI121-PutAMT1;1-GFP*, we observed that the GFP signal was predominantly localized in the plasma membrane (Figure 3A,B,C). Based on our observation, *PutAMT1;1-GFP* was noticeably localized in the plasma membrane and the nuclear periphery of yeast and *P. tenuiflora* cell suspensions (Figure 3D,E,F,G). The observed GFP fluorescence pattern of *PutAMT1;1-GFP* in the yeast cells was similar to that reported by Zhang *et al.*, (2011) [37]; however, current research indicates that all plant AMT1 proteins are localized in the plasma membrane [13,14,16,17].

**Figure 4:**
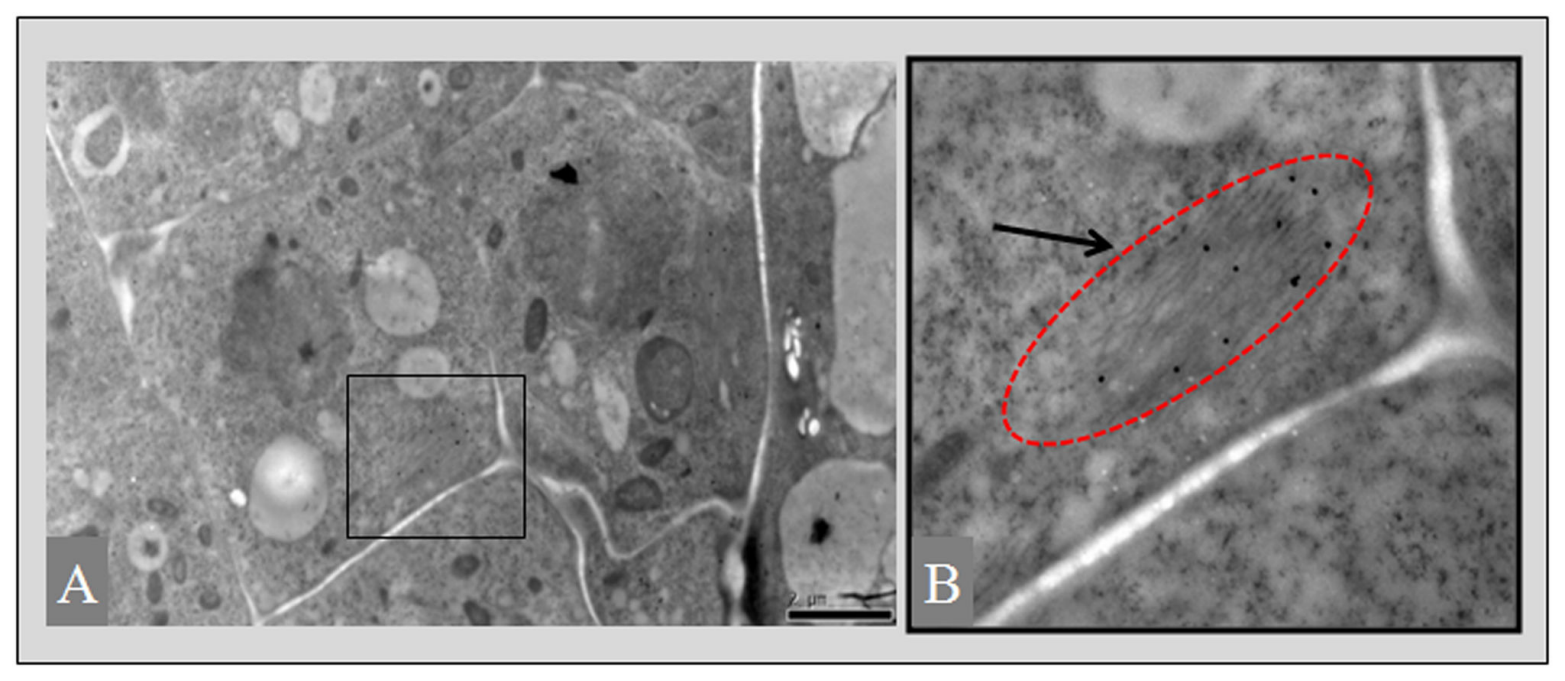
Immunogold localization of PutAMT1.1-GFP. Overview of an immunogold labeled cryosection of a root tip cortex cell expressing PutAMT1.1-GFP (A and B). *Arrows* indicate the gold particles that specifically labeled a thin layer of endomembrane. Scale bar is 1 µm. B is a magnified view of A.

To further understand the subcellular localization of PutAMT1;1-GFP in *Arabidopsis* cells, immunoelectron microscopy was performed. Gold particles specifically labeled a thin layer of endomembrane in ultra-thin cryosections of root tissue from PutAMT1;1-GFP seedlings incubated with a GFP antibody (Figure 4A,B). This result might indicate that PutAMT1;1 is localized in the endomembrane system of *Arabidopsis* cells.

### Functional complementation analysis of PutAMT1;1 in a yeast mutant strain

**Figure 5:**
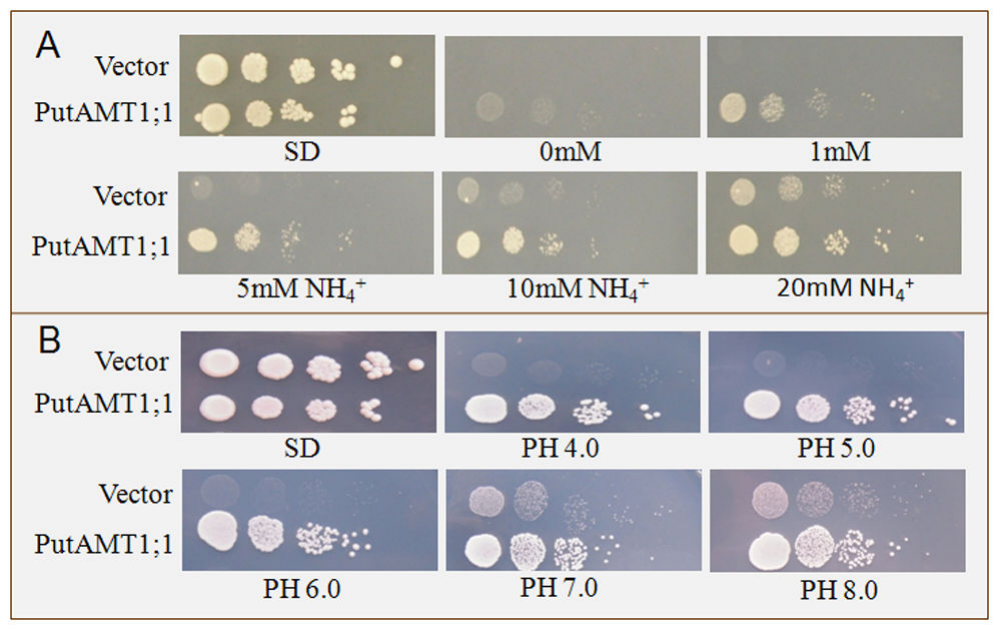
Growth complementation of the ammonium uptake-defective yeast strain 31019b by PutAMT1;1. The triple mep yeast mutant 31019b was transformed with the empty vector pYES2, pYES2-PutAMT1;1 growth; (A) on YNB medium containing either 0 mM, 1 mM, 5 mM, 10 mM, or 20 mM NH_4_^+^ as the sole nitrogen source in the presence of 2% (w/v) galactose, and (B) on different pH medium at 4.0, 5.0, 6.0, 7.0, and 8.0 in the presence of 1 mM NH_4_^+^ and 2% (w/v) galactose.

Transformation of 31019b with *pYES2-PutAMT1;1* allowed yeast growth on 1, 5, 10, or 20 mM (NH_4_)_2_SO_4_ as the sole nitrogen source, while the transformation of *pYES2* alone did not allow growth on 1 mM (NH_4_)_2_SO_4_ (Figure 5A). These results indicate that PutAMT1;1 complements the NH_4_^+^ uptake deficiency of 31019b; thus, the isolated cDNA of PutAMT1;1 encodes a functional high-affinity AMT, which is similar to previously identified AMT1 transporters [23,24]. In addition, the *pYES2-PutAMT1;1* transformant allowed yeast to grow under different pH conditions, ranging between 4.0 and 8.0; hence, *pYES-PutAMT1;1* function is not pH sensitive (Figure 5B).

### PutAMT1;1 transgenic plant response to different nitrogen sources

**Figure 6:**
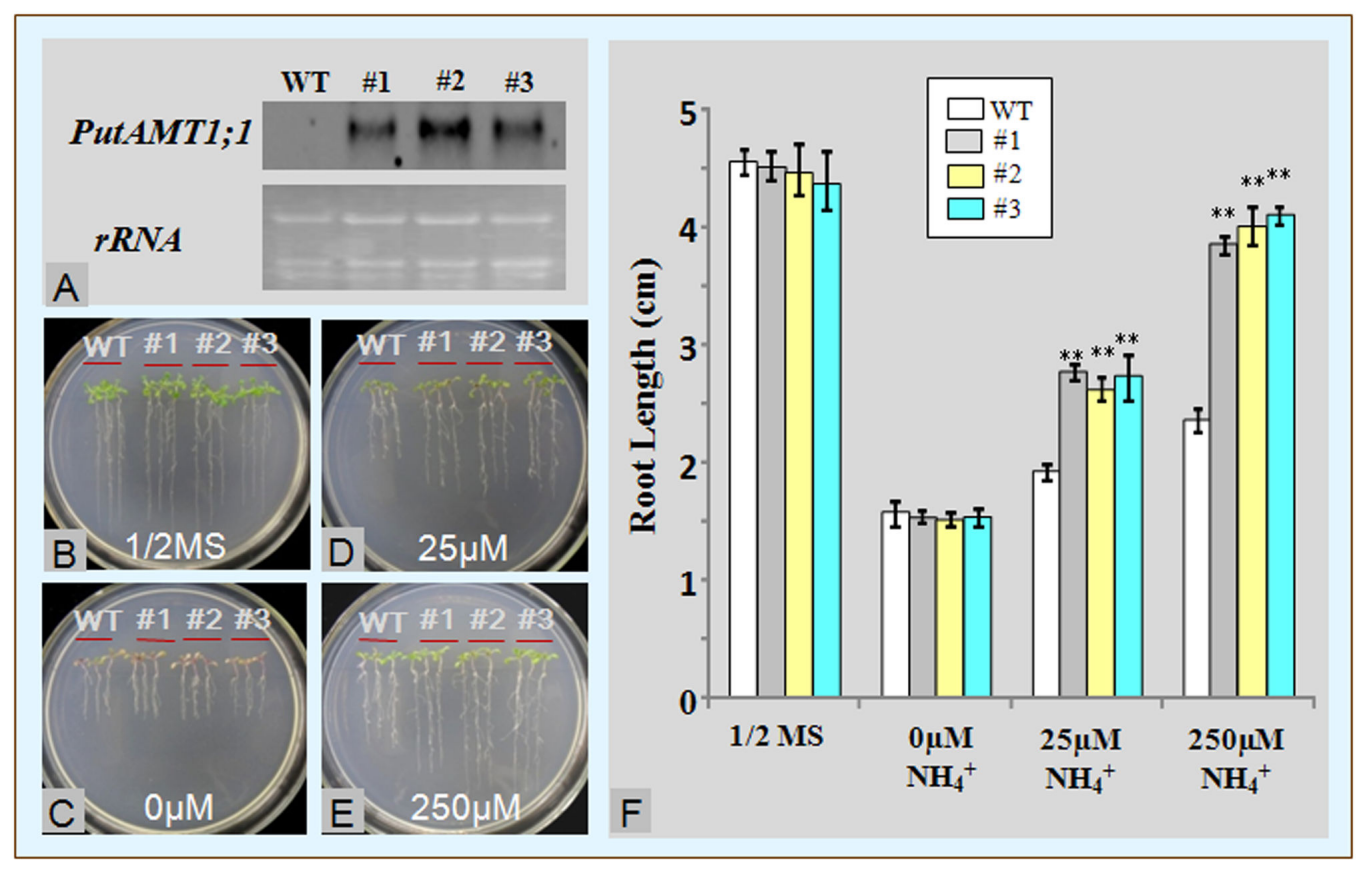
(A) RNA gel blot analysis of T3 transgenic plants expressing *PutAMT1;1*. WT: *Arabidopsis* (*Arabidopsis thaliana*) ecotype Columbia-0; #1, #2, and #3. T3 transferred Columbia-0 with PutAMT1;1. (B to F) Growth of wild-type and PutAMT1;1 transgenic lines on different amounts of nitrogen sources. Growth of WT plants and transgenic PutAMT1;1 plants (lines 1, 2, and 3) on agarose containing (B)1/2 MS, (C) 0 µM, (D) 25 µM, and (E) 250 µM ammonium succinate for 7 days after pre-culture on half-strength MS medium for 7 days. (F) Plot showing root growth vs. the amount of ammonium succinate. Bars indicate the means ± SE (n = 12). Statistical significance was determined using the Student’s *t*-test. * represents *p*<0.05 and ** represents *p*<0.01.

The RNA gel blot analysis results are shown as Figure 6A. The control samples had no signals, whereas the transgenic plants all expressed the gene; this result confirmed that the PutAMT1;1 gene has been successfully transformed. Thus, independent transgenic plants of PutAMT1;1 (#1, #2, #3) were used for the root length assays. Under 1/2MS and 0μM ammonium conditions, both wide-type and transgenic plants grew with no significant difference (Figure 6B,C). The 3 transgenic lines grew better than the wild-type in cultures containing 25 and 250 μM ammonium (Figure 6D,E). These results indicate that PutAMT1;1 mediates ammonium influx into plant roots. The observation of the PutAMT1:1 response at the lowest concentration of ammonium (25 μM) confirmed that it is a high affinity AMT. 

**Figure 7:**
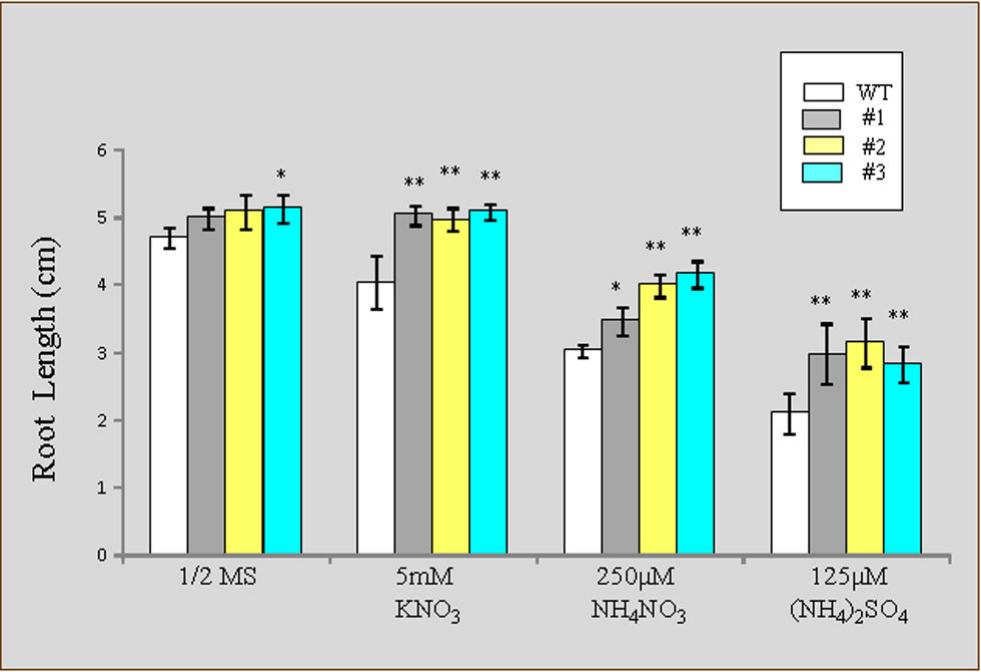
Growth of wild-type and PutAMT1;1 transgenic lines on different amounts of nitrogen sources. Root growth of WT plants and transgenic PutAMT1;1 plant (lines 1, 2, and 3) were plotted after incubating plants on agarose containing different nitrogen sources, including 5 mM KNO_3_, 250 µM NH_4_NO_3_, and 125 µM (NH_4_)_2_SO_4_ for 7 days, with plants being pre-cultured on half-strength MS medium for 7 days. Bars indicate means ± SE (n = 12). Statistical significance was determined using the Student’s *t*-test. * represents *p*<0.05 and ** represents *p*<0.01.

The growth of WT and transgenic PutAMT1;1 plants was also observed under different nutrient conditions. When all samples were precultured in half-strength MS medium for 7 days, followed by growth on agar containing 5 mM KNO_3_, 250 µM NH_4_NO_3_, and 125 µM (NH_4_)_2_SO_4_, the roots of the transgenic plants grew better compared to those of the WT plants under all conditions (Figure 7). Even in the presence of an exclusive source of nitrate, the length of transgenic plants roots was higher compared to the WT plants. This observation indicated that PutAMT1;1 overexpressors have higher nitrogen mobilization in the roots compared to the shoots.

**Figure 8:**
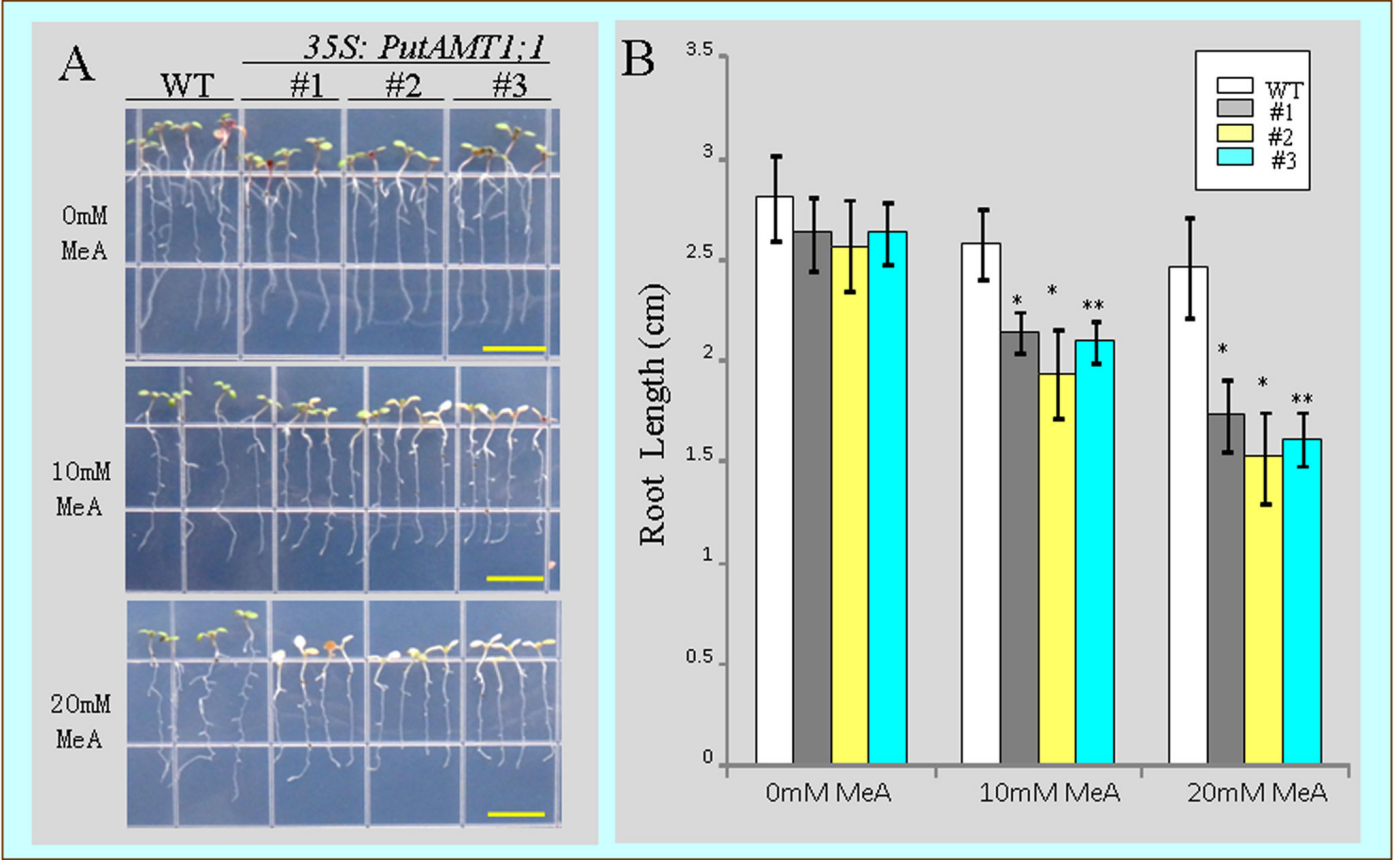
Growth of wild-type and *PutAMT1;1* transgenic lines on ammonium toxic analog methylammonium (MeA). (A) Growth of WT plants and transgenic *PutAMT1;1* plant (lines 1, 2, and 3) on agarose containing 0 mM, 10 mM or 20 mM MeA for 10 days after being pre-cultured on half-strength MS medium for 7 days. (B) Root length of the same plants as shown in (A). Bars indicate means ± SE (n = 12). Bars represent 1 cm. Statistical significance was determined using the Student’s *t*-test. * represents *p*<0.05 and ** represents *p*<0.01.

To test this hypothesis we assessed the effect of PutAMT1;1 on root to shoot transport. In addition to ammonium, plant AMT1-type transporter are also known to permeate the substrate analog MeA, which is toxic to plants [13,14]. In the transgenic lines, as MeA increased, leaves become noticeably bleached (discolored) and root growth declined (Figure 8). Therefore, transgenic plants were more sensitive to MeA compared to WT plants. These results indicate that PutAMT1;1 mediates MeA uptake from the medium to the roots, with the substance then being transported to the shoot. 

## Discussion

In this study, we characterized PutAMT1;1, which is a putative ammonium transporter found in the wild grass *P. tenuiflora*. Several lines of evidence clearly indicated that this gene codes as a functional AMT type1 gene: (1) the gene forms a well-supported and monophyletic clade with other AMT1 genes that have been isolated so far from representative monocotyledonous species, and (2) the predicted number and orientation of transmembrane domains exactly match those described for the other known plant AMT1 proteins. However, the localization of PutAMT1;1 in yeast and plant cells differed from other AMT1 transporter proteins previously found localized in the plasma membrane [13,14,16,17]. In addition, PutAMT1;1 was also observed in the nuclear periphery. Furthermore, the current study also indicates that the PutAMT1;1 protein might be localized in the endomembrane, e.g., the endoplasmic reticulum (ER). However, it was unclear as to whether PutAMT1;1 had a different function to other AMT1 proteins, due to its slightly different subcellular localization.

The current study confirmed that PutAMT1;1 is able to functionally complement the yeast strain 31019b, which is deficient in both the high- and the low-affinity ammonium transport systems [30]. Hence, PutAMT1;1 is the first AMT1 transporter of *P. tenuiflora* that has been characterized The closest homologue of PutAMT1;1 is TaAMT1;1 originating from *Triticum aestivum*; however, this transporter has not been characterized in detail, and only the fact that it is a high-affinity transporter, the activity of which seems to be largely pH-independent, has been reported [38]. The data assimilated here also indicate that PutAMT1;1 is a high-affinity AMT transporter [39], exhibiting largely pH-independent activity. However, the complete lack of information about the expression pattern of the AMT genes isolated from wheat prevents us from being able to conclude whether PutAMT1;1 is orthologous to TaAMT1;1. The next most closely related homologues are the 3 AMT1 transporters identified from the monocot rice (OsAMT1;1–3) [21,23]. PutAMT1;1 expression most closely resembles that of OsAMT1;1, which is constitutively expressed in both the root and shoot. Furthermore, both the ammonium inducibility of PutAMT1;1 and its reversibility closely match that of OsAMT1;1 in the root [23]. The slight inducibility of PutAMT1;1 in the shoot following nitrate treatment is another feature that it shares with OsAMT1;1. This activity was detected in the current study but not in the study by Sonoda and colleagues. This difference possibly arose because of the lower sensitivity of Southern blotting compared to real-time RT-PCR [40,41]. However, shoot PutAMT1;1 seems to be slightly repressed by ammonium at 2 h after treatment, which differs to that observed for OsAMT1;1. However, the effect of ammonium reversed to that of induction 1 day after treatment. This result indicates that temporal differences may exists in the regulation of the *P. tenuiflora* transporter compared to that of the rice homologue. Differences in the expression patterns observed for the AMT1 homologs have also been observed in other species (e.g., LjAMT1;1–3 from *Lotus japonicas* [42,43]; LeAMT1;1–3 from *Lycopersicon esculentum* [36]; AtAMT1;1–5 from *Arabidopsis* [4,24,44] ). Hence, the specialization of a single locus might be a common phenomenon in the regulation of ammonium transporters in plants [14]. Interestingly, PutAMT1;1 had a similar preferential expression in the reproductive organs with AtAMT1;4; however, this expression is not exclusive to the *Arabidopsis* homologue. Therefore, it might be possible that PutAMT1;1 is also involved in the symplasmic import of nitrogen to pollen [24]. Alternatively, the observed differences with the expression pattern of rice homologues might be due to the adaptation of *P. tenuiflora* to alkali soils, where nitrate is the dominant form of nitrogen available for plant growth [27]. A more precise characterization of the tissue-specific expression of PutAMT1;1, in addition to the number and expression pattern of other AMT homologues that have yet to be identified in *P. tenuiflora*, is required to confirm this hypothesis.

To further our understanding about the features of the PutAMT1;1 transporter, given the difficulties in transforming *P. tenuiflora* (S Liu, unpublished data), we also carried out a functional analysis of transgenic *Arabidopsis* plants overexpressing PutAMT1;1. The roots of plants from transgenic lines consistently grew larger than WT in solutions with reduced ammonium concentrations compared to MS medium (Figure 6E). This pattern was maintained irrespective of the nitrogen source provided (ammonium, nitrate, or a combination of the two; Figure 7). Due to the higher nitrogen absorption efficiency of transgenic plants, which also arose in non-nitrogen-limiting conditions (1/2 MS medium), the roots of transgenic plants also grew larger than WT plants after a certain period of culturing. This phenomenon arose because nitrate absorbed from the roots is reduced to ammonia before it is mobilized to the shoots [27]. Overall, these results indicate the capacity of PutAMT1;1 to enhance root to shoot nitrogen mobilization in overexpressing lines. This hypothesis was further supported by the fact that the administration of increasing MeA concentrations to the growth medium caused progressively stronger toxicity symptoms in the shoot of overexpressed lines compared to that of WT (Figure 8). This result is consistent with previous studies demonstrating that AMT1 transporters do not differentiate between ammonium and its toxic analog MeA [16,17]. Previous studies have indicated that OsAMT1;1 plays a major role in the nitrogen-use efficiency of different rice cultivars, which have been demonstrated to differentially express this high affinity nitrogen transporter in the leaves [45]. Furthermore, it has been proposed that the high-affinity nitrogen transporter OsAMT1;1 in the aerial tissues of rice might be involved in the enhancement of NH_4_^+^ being downloaded from the xylem [45]. On the basis of these results, a possible mechanism accounting for the observed growth enhancement of transgenic *Arabidopsis* might be a higher sink strength of individual cells over-expressing PutAMT1;1. According to this model, by increasing the number of cells competent for high-affinity nitrogen uptake, the overall sink strength of the plant would be enhanced. At the shoot level, this activity would result in higher vascular download, and the enhancement of the overall root-to-shoot nitrogen mobilization. At the root level, this activity would also result in higher uptake capacity from the soil. However, at present, the reason for the higher growth of roots compared to aerial parts in the transgenic lines is not clear, with further research being required to elucidate this phenomenon.

With the increasing demand for food production, due to worldwide population growth, the need for more efficient nitrogen uptake and utilization by important crop species, like rice, is becoming an economically and socially relevant priority. However, the majority of the studies on high-efficiency nitrogen transport focus on dicotyledonous species, with the AMT1 genes of monocots remaining poorly characterized from a functional point of view [46]. The characterization of the first AMT1 transporter from the wild species *P. tenuiflora* is expected to help further our understanding about the process of nitrogen assimilation by plants. Hoque (2006) reported that transgenic rice plants constitutively expressing OsAMT1;1 under the control of the maize ubiquitin promoter displayed an augment rate of NH_4_^+^ depletion from the nutrient solution, accompanied by an increment of NH_4_^+^ concentrations in the roots and shoots; however, transgenic plant growth was retarded compared to WT growth [47]. The unique pattern of expression observed for PutAMT1;1 might indicate a different regulation/function compared to the rice homologues identified so far. It might be speculated that such differences reflect adaptations specific to the low ammonium availability in the alkali-saline soils where *P. tenuiflora* is one of the dominant species in northern China. If so, the promoter and/or the coding regions of PutAMT1;1 might represent valid candidates for improving the performance of crop species subject to nitrogen-limiting conditions. Even when overexpressed in *Arabidopsis*, our data showed an improvement of root to shoot nitrogen mobilization when mediated by PutAMT1;1. Therefore, if this phenomenon could be replicated in rice, PutAMT1;1 might represent a promising tool to improve nitrogen utilization by monocot crops grown under nutrient-deficient conditions [46].

## References

[1] Coruzzi G, Bush DR (2001) Nitrogen and carbon nutrient and metabolite signaling in plants. Plant Physiol 125: 61-64. doi:10.1104/pp.125.1.61. PubMed: 11154297. 11154297 PMC1539326

[2] Judkins G, Myint S (2012) Spatial Variation of Soil Salinity in the Mexicali Valley, Mexico:Application of a Practical Method for Agricultural Monitoring. Environ Manage 50: 478–489. doi:10.1007/s00267-012-9889-3. PubMed: 22744157. 22744157

[3] Yuan ZQ (2010) Analysis of Saline-Alkali characteristics and nutrient status of soil in Western Jilin Province. Dissertation, Northeast Normal University, China.

[4] Gazzarrini S, Lejay L, Gojon A, Ninnemann O, Frommer WB et al. (1999) Three functional transporters for constitutive, diurnally regulated, and starvation-induced uptake of ammonium into *Arabidopsis* roots. Plant Cell 11: 937-947. doi:10.1105/tpc.11.5.937. PubMed: 10330477. 10330477 PMC144234

[5] Bloom AJ, Sukrapanna SS, Warner RL (1992) Root respiration associated with ammonium and nitrate absorption and assimilation by barely. Plant Physiol 99: 1294-1301. doi:10.1104/pp.99.4.1294. PubMed: 16669035. 16669035 PMC1080623

[6] Britto DT, Siddiqi MY, Glass ADM, Kronzucker HJ (2001) Futile transmembrane NH_4_ ^+^ cycling: A cellular hypothesis to explain ammonium toxicity in plants. Proc Natl Acad Sci U S A 98: 4255-4258. doi:10.1073/pnas.061034698. PubMed: 11274450. 11274450 PMC31212

[7] Kronzucker HJ, Britto DT, Davenport RJ, Tester M (2001) Ammonium toxicity and the real cost of transport. Trends Plant Sci 6: 335-337. doi:10.1016/S1360-1385(01)02022-2. PubMed: 11495764. 11495764

[8] Szczerba MW, Britto, Ali SA, Balkos KD, Kronzucker HJ (2008) NH_4_ ^+^ stimulated and -inhibited components of K+ transport in rice (*Oryza* *sativa* L). J Exp Bot 59: 3415-3423 10.1093/jxb/ern190 PMC2529248 18653690

[9] Schachtman DP, Schroeder JI, Lucas WJ, Anderson JA, Gaber RF (1992) Expression of an inward-rectifying potassium channel by the *Arabidopsis* KAT1 cDNA. Science 258: 1654-1658. doi:10.1126/science.8966547. PubMed: 8966547. 8966547

[10] Nakhoul NL, Hering-Smith KS, Abdulnour-Nakhoul SM, Hamm LL (2001) Transport of NH_3_/NH_4_ ^+^ in *oocytes* expressing aquaporin-1. Am J Renal Physiol 281(2): 255-263. 10.1152/ajprenal.2001.281.2.F255 11457716

[11] Holm LM, Jahn TP, Møller A, Schjoerring JK, Ferri D et al. (2005) NH_3_ and NH_4_ ^+^ permeability in aquaporin-expressing *Xenopuso* *ocytes* . Pflugers Arch 450: 415-428. doi:10.1007/s00424-005-1399-1. PubMed: 15988592. 15988592

[12] Loqué D, Ludewig U, Yuan L, von Wirén N (2005) Tonoplastintrinsic proteins AtTIP2;1 and AtTIP2;3 facilitate NH_3_ transport into the vacuole. Plant Physiol 137: 671-680. doi:10.1104/pp.104.051268. PubMed: 15665250. 15665250 PMC1065367

[13] Loqué D, Yuan L, Kojima S, Gojon A, Wirth J et al. (2006) Additive contribution of AMT1;1 and AMT1;3 to high-affinity ammonium uptake across the plasma membrane of nitrogen-deficient *Arabidopsis* roots. Plant J 48: 522-534. doi:10.1111/j.1365-313X.2006.02887.x. PubMed: 17026539. 17026539

[14] Yuan L, Loqué D, Kojima S, Rauch S, Ishiyama K et al. (2007) The organization of high-affinity ammonium uptake in *Arabidopsis* roots depends on the spatial arrangement and biochemical properties of AMT1-type transporters. Plant Cell 19: 2636-2652. doi:10.1105/tpc.107.052134. PubMed: 17693533. 17693533 PMC2002620

[15] Loqué D, von Wirén N (2004) Regulatory levels for the transport ammonium in plant roots. J Exp Bot 55: 1293-1305. doi:10.1093/jxb/erh147. PubMed: 15133056. 15133056

[16] Ludewig U, von Wirén N, Frommer WB (2002) Uniport of NH_4_ ^+^ by the root hair plasma membrane ammonium transporter LeAMT1;1. J Biol Chem 277: 13548-13555. doi:10.1074/jbc.M200739200. PubMed: 11821433. 11821433

[17] Ludewig U, Wilken S, Wu B, Jost W, Obrdlik P et al. (2003) Homo-and hetero-oligomerization of ammonium transporter-1 NH_4_ ^+^ uniporters. J Biol Chem 278: 45603-45610. doi:10.1074/jbc.M307424200. PubMed: 12952951. 12952951

[18] Mayer M, Dynowski M, Ludewig U (2006) Ammonium iontransport by the AMT/Rh homologue LeAMT1;1. Biochem J 396: 431-437. doi:10.1042/BJ20060051. PubMed: 16499477. 16499477 PMC1482825

[19] Howitt SM, Udvardi MK (2000) Structure, function and regulation of ammonium transporters in plants. BBA-Biomembranes 1465:152-170. 10.1016/s0005-2736(00)00136-x 10748252

[20] Sohlenkamp C, Shelden M, Howitt S, Udvardi MK (2000) Characterization of *Arabidopsis* AtAMT2, a novel ammonium transporter in plants. FEBS Lett 467: 273-278. doi:10.1016/S0014-5793(00)01153-4. PubMed: 10675553. 10675553

[21] Kumar A, Silim SN, Okamoto M, Siddiqi MY, Glass ADM (2003) Differential expression of three members of the AMT1 gene family encoding putative high-affinity NH_4_ ^+^ transporter in roots of *Oryza* *sativa* subspecies indica. Plant Cell Environ 26: 907-914. doi:10.1046/j.1365-3040.2003.01023.x. PubMed: 12803618. 12803618

[22] Rawat SR, Silim SN, Kronzucker HJ, Siddiqi MY, Glass AD (1999) AtAMT1 gene expression and NH_4_ ^+^ uptake in roots of *Arabidopsis* . Plant Journal 19: 143-152. doi:10.1046/j.1365-313X.1999.00505.x. PubMed: 10476061. 10476061

[23] Sonoda Y, Ikeda A, Saiki S, von Wirén N, Yamaya T et al. (2003) Distinct expression and function of three ammonium transporter genes (OsAMT1;1–1;3) in rice. Plant Cell Physiol 44: 726-734. doi:10.1093/pcp/pcg083. PubMed: 12881500. 12881500

[24] Yuan L, Graff L, Loqué D, Kojima S, Tsuchiya YN et al. (2009) AtAMT1;4, a pollen-specific high-affinity ammonium transporter of the plasma membrane in *Arabidopsis* . Plant Cell Physiol 50: 13-25. doi:10.1093/pcp/pcn186. PubMed: 19073648. 19073648 PMC2638712

[25] Sohlenkamp C, Wood CC, Roeb GW, Udvardi MK (2002) Characterization of *Arabidopsis* AtAMT2, a high-affinity ammonium transporter of the plasma membrane. Plant Physiol 130: 1788-1796. doi:10.1104/pp.008599. PubMed: 12481062. 12481062 PMC166690

[26] Simon-Rosin U, Wood C, Udvardi MK (2003) Molecular and cellular characterisation of LjAMT2;1, an ammonium transporter from the model legume *Lotus* *japonicus* . Plant Mol Biol 51 : 99-108. doi:10.1023/A:1020710222298. PubMed: 12602894 . 12602894

[27] Wang H, Wu Z, Han J, Zheng W, Yang C (2012) Comparison of ion balance and nitrogen metabolism in old and young leaves of alkali-stressed rice plants. PLOS ONE 7(5): e37817. doi:10.1371/journal.pone.0037817. PubMed: 22655071. 22655071 PMC3360002

[28] Wang ZQ, Zhu SQ, Yu RP, Li LQ, Shan GZ (1993) The saline alkali soil of China. The Science Press, Beijing, China.

[29] Shi DC, Yin LJ (1993) Difference between salt (NaCl) and alkaline (Na_2_CO_3_) stresses on *Pucinellia* *tenuiflora* (Griseb.) Scribn. et Merr. plants. Acta Bot Sin 35: 144–149 (in Chinese with English abstract)

[30] Marini AM, Soussi-Boudekou S, Vissers S, Andre B (1997) A family of ammonium transporters in *Saccharomyces* *cerevisiae* . Mol Cell Boil 17: 4282-4293. 10.1128/mcb.17.8.4282 PMC232281 9234685

[31] Kumar S, Tamura K, Nei M (2004) MEGA3: Integrated software for Molecular Evolutionary Genetics Analysis and sequence aligament. Brief Bioinform 5: 150-163. doi:10.1093/bib/5.2.150. PubMed: 15260895. 15260895

[32] Gietz RD, Schiestl RH, Willems AR, Woods RA (1995) Studies on the transformation of intact yeast cells by the LiAc/SS-DNA/PEG procedure. Yeast 11: 355-360. doi:10.1002/yea.320110408. PubMed: 7785336. 7785336

[33] Sheen J (2002) A transient expression assay using *Arabidopsis* mesophyII protoplasts. Available: http://genetics.mgh.harvard.edu/sheenweb/.

[34] Sambrook J, Fritsch EF, Maniatis T (1989) Molecular Cloning: A Laboratory Manual, 2nd. New York: Cold Spring Harbor Laboratory Press pp.1566–1573.

[35] Clough SJ, Bent AF (1998) Floral dip: a simplified method for Agrobacterium-mediated transformation of *Arabidopsis* *thaliana* . Plant J 16: 735-743. doi:10.1046/j.1365-313x.1998.00343.x. PubMed: 10069079. 10069079

[36] von Wirén N, Lauter FR, Ninnemann O, Gillissen B, Walch-Liu P et al. (2000) Differential regulation of three functional ammonium transporter genes by nitrogen in root hairs and by light in leaves of tomato. Plant J 21: 167-175. doi:10.1046/j.1365-313x.2000.00665.x. PubMed: 10743657. 10743657

[37] Zhang X, Zhang M, Takano T, Liu S (2011) Characterization of an *AtCCX5* gene from *Arabidopsis* *thaliana* that involves in high-affinity K^+^ uptake and Na^+^ transport in yeast. Biochemical and Biophysical Research Commun 414: 96-100. doi:10.1016/j.bbrc.2011.09.030. 21945443

[38] Jahn TP, Møller AL, Zeuthen T, Holm LM, Klærke D et al. (2004) Aquaporin homologues in plants and mammals transport ammonia. FEBS Lett 574: 31–36. doi:10.1016/j.febslet.2004.08.004. PubMed: 15358535. 15358535

[39] Wang MY, Siddiqi MY, Ruth TJ, Glass DM (1993) Ammonium uptake by rice roots. II. Kinetics of 13NH_4_ ^+^ influxes across the plasmalemma. Plant Physiol 103: 1259-1267. PubMed: 12232018. 12232018 10.1104/pp.103.4.1259 PMC159114

[40] Zhao XQ, Zhao SP, Shi WM (2008) Enhancement of NH_4_ ^+^ uptake by NO_3_ ^-^ in relation to expression of nitrate-induced gene in rice (*Oryza* *sativa* L.) roots. Pedosphere 18: 86-91. doi:10.1016/S1002-0160(07)60106-4.

[41] Duan YH, Yin XM, Zhang YL, Shen QR (2007) Mechanisms of the enhanced rice growth and nitrogen uptake by nitrate. Pedosphere 17: 697-705. doi:10.1016/S1002-0160(07)60084-8.

[42] Salvemini F, Marini AM, Riccio A, Patriarca EJ, Chiurazzi M (2001) Functional characterization of an ammonium transporter gene from *Lotus* *japonicas* .Gene 270: 237-243. doi:10.1016/S0378-1119(01)00470-X. PubMed: 11404021. 11404021

[43] D'Apuzzo E, Rogato A, Simon-Rosin U, El Alaoui H, Barbulova A et al. (2004) Characterization of three functional high-affinity ammonium transporters in *Lotus* *japonicus* with differential transcriptional regulation and spatial expression. Plant Physiol 134: 1763-1774. doi:10.1104/pp.103.034322. PubMed: 15075393. 15075393 PMC419849

[44] Ninnemann O, Jauniaux JC, Frommer WB (1994) Identification of a high affinity NH_4_ ^+^ transporter from plants. EMBO J 13: 3464-3471. PubMed: 8062823. 8062823 10.1002/j.1460-2075.1994.tb06652.x PMC395249

[45] Gaur VS, Singh US, Gupta AK, Kumar A (2012) Influence of different nitrogen inputs on the members of ammonium transporter and glutamine synthetase genes in two rice genotypes having differential responsiveness to nitrogen. Mol Biol Rep 39: 8035-8044. doi:10.1007/s11033-012-1650-8. PubMed: 22531935. 22531935

[46] Vinod KK, Heuer S (2012) Approaches towards nitrogen- and phosphorus-efficient rice. AoB PLANTS pls028 . 10.1093/aobpla/pls028 PMC3484362 23115710

[47] Hoque MS, Masle J, Udvardi MK, Ryan PR, Upadhyaya NM (2006) Over-expression of the rice OsAMT1-1 gene increases ammonium uptake and content, bur impairs growth and development of plants under high ammonium nutrient. Funct Plant Biol 33: 153-163. 10.1071/FP05165 32689222

